# 

**Published:** 1886-08

**Authors:** 


### BOOKS RECEIVED.

*Reports of the Surgeon-General of the Navy.* Six volumes, 1879, '80, '81, '82, '83, '84.

*Hygienic and Medical Reports United States Navy 1879.*

*Traite Elementaire d'Anatomie Medicale du Systeme Nerveux.* Par Ch. Tere. Paris: Bureaux du Progres Medical.

*Our Penal Machinery and Its Victims.* By John P. Altgeld. A. C. McClurg & Co., Chicago.

*A Treatise on the Diseases of the Nervous System.* By William A. Hammond, M. D. New York: D. Appleton & Co. Chicago: A. C. McClurg & Co.

*Medicine of the Future.* By Austin Flint (Senior), M. D., LL. D. New York: D. Appleton & Co. Chicago: A. C. McClurg & Co.

*Analysis of the Urine.* By Hoffman & Ultzmaun. New York: D. Appleton & Co. Chicago: A. C. McClurg & Co.

*Bright's Disease and Allied Affections of the Kidneys.* By Charles W. Purdy. Philadelphia: Lea Brothers & Co. Chicago: A. C. McClurg & Co.

*Medical and Surgical Directory of the United States.* R. L. Polk & Co.

### PAMPHLETS RECEIVED.

*Report of the Board of Managers of the Pennsylvania Hospital 1886.*

*Is Disease of theUterine Appendages as Frequent as It Has Been Reported?* By Henry C. Coe, M. D.

*The Influence of Sewerage and Water Supply on the Death Rate in Cities.* By Erwin F. Smith.

*Report of a Case of Cæsarean Operation, with Some Comments.* By Edward W. Jenks, M. D.

*Typhoid Fever in Philadelphia.* By Henry Leffmann, M. D.

*Tetanus.* By N. Senn, M. D.

*Comments on Pasteur's Method of Treating Hydrophobia.* By Charles W. Dulles, M. D.

*On the Necessity of Organization of the Medical Profession.* By F. E. Daniel, M. D.

*On the Limitation of the Contagious Stage of Syphilis.* By F. N. Otis, M. D.

*American Public-Health Association. Preliminary Circular.*

*The Present Status of Abdominal Surgery.* By N. Senn, M. D.

*Excerpta from the Biennial Report of the Board of Health of Louisiana.*

*A Brief Synopsis of the Various Points Involved in the Coarse Examination of the Brain and Spinal Cord.* By Francis X. Dercum, M. D.

*Malarial Manifestations Due to Traumatism.* By Henry C. Coe, M. D., M. R. C. S.

*Esthetics of Medicine.* By H. A. Cottell, M. D.

*Enucleation with Transplantation and Reimplantation of Eyes.* By Charles H. May, M. D.

*Marion County, Florida; an Ideal Winter Climate.* By George Troup Maxwell, M. D.

*Report of a Case of Successful Transfusion in Typhoid Fever.* By W. S. Whitwell, A. M., M.D.

SUBSCRIPTION PRICE, $3.00 PER YEAR.

Whole Number, 319. AUGUST, 1886. Vol. LIII. Number 2.

LIBRARY
INDEXED
S.G.O.

THE

### CHICAGO MEDICAL JOURNAL AND EXAMINER

EDITORS:

S. J. JONES, M.D., LL.D.

W. W. JAGGARD, A.M., M.D. HAROLD N. MOYER, M.D.

COLLABORATORS.

CHICAGO:

PROFESSOR J. A. ALLEN, M. D., LL. D.

PROFESSOR E. ANDREWS, M. D., LL. D.

PROFESSOR W. H. BYFORD, A. M., M. D.

PROFESSOR O. C. DEWOLF, A. M., M. D.

PROFESSOR E. C. DUDLEY, A. B., M. D.

PROFESSOR C. FENGER, M. D.

PROFESSOR J. N. HYDE, A. M., M. D.

PROFESSOR E. F. INGALS, A. M., M.D.

PROFESSOR H. A. JOHNSON, M. D., LL. D.

CHARLES GILMAN SMITH, A. M., M.D.

PROFESSOR J. H. ETHERIDGE, A. M., M. D.

MILWAUKEE, WISCONSIN:

PROFESSOR N. SENN, M. D.

WASHINGTON, D. C.:

S. O. RICHEY, M. D.

UNITED STATES ARMY:

SURGEON, D. L. HUNTINGTON, M. D.

UNITED STATES NAVY:

MEDICAL DIRECTOR, J. M. BROWNE, M. D.

MEDICAL DIRECTOR, A. L. GIHON, A. M., M. D.

